# Left Upper Division Segmentectomy Compared with Lobectomy for Lung Expansion and Bronchus Tortuosity

**DOI:** 10.1245/s10434-024-15012-6

**Published:** 2024-04-01

**Authors:** Wongi Woo, Chul Hwan Park, Jimin Lee, Duk Hwan Moon, Sungsoo Lee

**Affiliations:** 1 Department of Thoracic and Cardiovascular Surgery, Gangnam Severance Hospital, Yonsei University College of Medicine, Seoul, Republic of Korea; 2 Department of Radiology, Gangnam Severance Hospital, Yonsei University College of Medicine, Seoul, Republic of Korea

**Keywords:** Bronchial angle, Postoperative lung function, Left upper division segmentectomy, Left upper lobectomy, Non-small cell lung cancer, Propensity score matching

## Abstract

**Background:**

For patients with left upper lobe lesions, the functional benefit of left upper division segmentectomy over left upper lobectomy remains controversial. This study evaluated the clinical and functional outcomes after these two procedures.

**Methods:**

This retrospective study included 135 patients with left upper lobe lesions (left upper lobectomy, 110; left upper division segmentectomy, 25). Propensity score matching was used to compare the two groups. Spirometry and computed tomography volume assessments were performed to evaluate bronchus angle and tortuosity. Short-term clinical respiratory symptoms were assessed via medical record reviews.

**Results:**

Patients in both groups had similar preoperative characteristics, apart from tumor size (left upper division segmentectomy, 1.6 ± 0.9 cm; left upper lobectomy, 2.8 ± 1.7 cm; *p* = 0.002). After propensity score matching, both groups had similar preoperative spirometry and pathological results. The postoperative spirometry results were similar; however, the left upper division segmentectomy group had a significantly smaller decrease in left-side computed tomography lung volume compared with that in the left upper lobectomy group (left upper division segmentectomy, 323.6 ± 521.4 mL; left upper lobectomy, 690.7 ± 332.8 mL; *p* = 0.004). The left main bronchus-curvature index was higher in the left upper lobectomy group (left upper division segmentectomy, 1.074 ± 0.035; left upper lobectomy, 1.097 ± 0.036; *p* = 0.013), and more patients had persistent cough in the left upper lobectomy group (*p* = 0.001).

**Conclusions:**

Left upper division segmentectomy may be a promising option for preventing marked bronchial angulation and decreasing postoperative persistent cough in patients with left upper lobe lung cancer.

Due to advancements in surgical and lung cancer screening techniques, an increasing number of patients are diagnosed with early-stage lung cancer. Patients diagnosed with non-small cell lung cancer (NSCLC) at an early stage generally experience favorable postoperative outcomes. The surgical approach is determined based on various factors, including the risk profile, extent of cancer, and postoperative lung function or quality of life for the patient. Two recent randomized controlled trials, CALGB 140503 and JCOG 0802,^1,2^ compared the efficacy of sublobar resection and lobectomy and demonstrated that sublobar resection is an effective treatment modality for NSCLC. This technique has gained support from the surgical community owing to its ability to preserve lung parenchyma and pulmonary function.

For lesions in the left anterior and apical-posterior pulmonary segments (left upper division, [LUD]), LUD segmentectomy (LUDS) yields oncological outcomes comparable with those of lobectomy regarding recurrence-free and overall survival rates.^3–5^ However, evidence regarding the benefit of preserving the lingular segment in terms of postoperative pulmonary function is lacking.^6–8^ Despite lingular preservation, the lingular segment may have significantly smaller volume relative to that of the LUD and may not function properly after surgery. Furthermore, the benefits of segmentectomy primarily involve one or two segmental resections, which result in improved preservation of parenchymal volume. As the LUD is comparable in size to the right upper lobe, resecting multiple segments, such as the LUD, could significantly decrease pulmonary function beyond expectation. Considering the lack of clarity regarding the benefits of LUD resection and lung function in comparison with those of left upper lobectomy (LUL), performing a lobectomy may be more suited to achieve a larger oncological resection margin.

Currently, most methods used to evaluate postoperative pulmonary function rely heavily on the results of spirometry, which is the most validated and clinically practical technique. However, clinicians may encounter several issues when comprehensively evaluating postoperative pulmonary function using spirometry. Lu et al.^9^ reported that bronchial kinking after right upper lobectomy, assessed by measuring the angle between the right bronchus intermedius and lower lobe bronchus, may be associated with postoperative respiratory symptoms. Furthermore, morphological changes in the bronchi are also associated with the degree of reduction in the forced expiratory volume in 1 s (FEV_1_).^10^ We previously demonstrated that preservation of the inferior pulmonary ligaments affected bronchial tortuosity^11^; however, reports on comprehensive examinations for the effects of bronchial angle or tortuosity are limited. Thus, in this study, we evaluated the change in the angle of the left main bronchus after LUDS/LUL and investigated its impact on postoperative respiratory symptoms.

## Methods

### Ethical Statement

This study was approved by the Institutional Review Board of Gangnam Severance Hospital, Yonsei University College of Medicine (No. 3-2023-1247, 03-01-2023). Informed consent was waived due to the retrospective nature of the study.

### Participants

In this retrospective study, we reviewed the records of patients diagnosed with stage I–II NSCLC between January 2014 and December 2021. Of these 1055 patients, we only included those with left upper lobe lesions (*n* = 244). Patients with stage III–IV disease (*n* = 22), wedge resection (*n* = 70), non-R0 resection (*n* = 5), and those without preoperative or 3-month postoperative pulmonary function tests (PFTs; *n* = 12) were excluded from the study. Finally, 135 patients (LUL, *n* = 110; LUDS, *n* = 25) were included in the study. To minimize the effect of confounding factors, demographic variables such as age, sex, and body mass index (BMI) were matched using propensity scores before the groups were compared.

### Chest Computed Tomography (CT) Analysis—Lung Volume Measurement

Three-dimensional lung volumes were semi-automatically measured using commercially available reconstruction software (Aquarius iNtuition™ Ver.4.4.6; TeraRecon, Foster City, CA, USA) according to a previously reported, threshold-based, three-dimensional auto-segmentation technique. Anatomical lung volume was measured using a density mask and auto-segmentation techniques. Voxels with Hounsfield Unit (HU) values ranging from −1024 to −200 HU were selected on the axial computed tomography (CT) images and their volume was measured semi-automatically.

### Chest CT Analysis—Bronchial Tortuosity Measurement

**Fig. 1:**
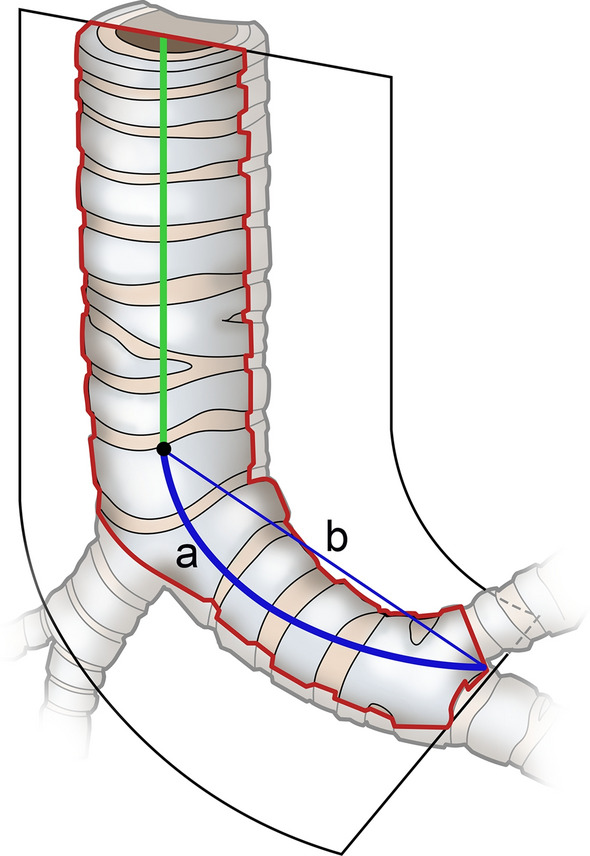
Measurement of left main bronchus tortuosity. The curvature index was used to measure the tortuosity of the left main bronchus. A center line passing through the distal trachea and left main bronchus was automatically created and carefully modified as needed. The distance of the curved center line from the carina to the second carina (**a**) and the distance of the straight line from the carina to the second carina (**b**) were used as the curvature index (**a, b**), representing the tortuosity of the left main bronchus. Higher indices indicated greater tortuosity

**Fig. 2:**
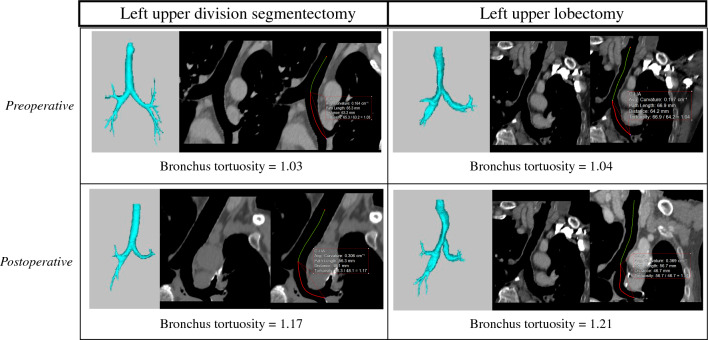
Representative CT images of left main bronchus tortuosity measurement in each group. *CT* computed tomography

Tortuosity of the left main bronchus was defined as the curvature index (CI), i.e. the curved length of the left main bronchus divided by the straight length of the left main bronchus (Fig. 1). Using commercially available reconstruction software, the full length of the left main bronchus was semi-automatically traced using the curved multiplanar technique. The CI was calculated as the midline curve length of the left main bronchus divided by the straight length measured using an electronic caliper after precise manual correction. Figure 2 demonstrates the representative images of measuring left main bronchus tortuosity in each group.

### Follow-Up Protocols

Patients were regularly followed up within 2 weeks of discharge from surgery. Subsequently, they visited the outpatient clinics at 1, 3, 6, and 12 months. Patients with respiratory symptoms were advised to visit the clinics every 2 weeks, and those with persistent respiratory symptoms were mutually assessed by pulmonologists and thoracic surgeons at the outpatient clinic. For patients with dyspnea, cardiogenic causes were also assessed using cardiac evaluations such as echocardiography. Persistent cough was defined as a cough lasting over 3 weeks after excluding other respiratory causes based on laboratory and radiologic evaluations.

### Statistical Analyses

For continuous variables, data are presented as mean and standard deviation. Data with normal distribution were analyzed using the two-sample *t*-test, and Fisher’s exact test was used to compare categorical variables. Propensity score matching (PSM) was applied to minimize the effect of confounding factors before comparing the clinical variables between the LUL and LUDS groups. The propensity score for each participant was calculated using a logistic model that included age, sex, and BMI. Statistical analyses were performed using R version 4.0.4 (R Core Team, R Foundation for Statistical Computing, Vienna, Austria). Differences with a two-tailed *p* value <0.05 were considered statistically significant.

## Results

### Clinical Characteristics After Propensity Score Matching

Patients in the LUL and LUDS groups had similar preoperative characteristics (Table 1); however, the LUL group had significantly larger tumors (LUDS, 1.6 ± 0.9 cm; LUL, 2.8 ± 1.7 cm; *p* = 0.002) and included more patients with stage II NSCLC (*p* = 0.002). Although the follow-up duration was not sufficiently long, no significant differences were observed in mortality or recurrence.

**Table 1:** Clinical characteristics of patients with left-side NSCLC according to the extent of surgical resection

Clinical variables		LUDS	LUL	*p* value
		[*n* = 25]	[*n* = 110]	
Age, years		61.6 ± 11.9	63.6 ± 10.8	0.423
Sex	Female	16/25 (64.0)	48/110 (43.6)	0.078
	Male	9/25 (36.0)	62/110 (56.4)	
BMI, kg/m^2^		25.2 ± 4.5	24.7 ± 3.1	0.462
Smoking history	Never	18/25 (72.0)	66/110 (60.0)	0.149
	Ex or current	7/25 (28.0)	44/110 (40.0)	
Diabetes mellitus		2/25 (8.0)	13/110 (11.8)	0.733
Hypertension		12/25 (48.0)	42/110 (38.2)	0.490
Cardiovascular disease		4/25 (16.0)	13/110 (11.8)	0.739
COPD		0/25 (0.0)	2/110 (1.8)	1.000
FEV_1_, L		2.33 ± 0.73	2.54 ± 0.62	0.169
FEV_1_/FVC, %		73.7 ± 10.4	79.5 ± 62.3	0.666
DLCO/VA, %		104.7 ± 14.7	105.9 ± 20.6	0.791
Surgical approach	Thoracotomy	17/25 (68.0)	67/110 (60.9)	0.649
	VATS	8/25 (32.0)	43/110 (39.1)	
*Histopatholgoic outcome*				
Tumor size, cm		1.6 ± 0.9	2.8 ± 1.7	0.002
Cell type	Squamous cell carcinoma	0 (0.0)	14/110 (12.7)	0.235
	Adenocarcinoma (non-mucinous)	25/25 (100.0)	88/110 (80.0)	
	Large cell carcinoma	0 (0.0)	1/110 (0.9)	
	Small cell carcinoma	0 (0.0)	2/110 (1.8)	
	Others	0 (0.0)	5/110 (4.5)	
LVI		0/25 (0.0)	13/110 (11.8)	0.126
VPI		1/23 (4.3)	18/97 (23.7)	0.116
Perineural invasion		0/25 (0.0)	1/110 (1.0)	1.000
EGFR mutation	Negative	3/20 (15.0)	14/70 (20.0)	0.754
	Positive	17/20 (85.0)	56/70 (80.0)	
Stage	I	23/25 (92.0)	65/110 (59.0)	0.002
	II	2/25 (8.0)	45 (41.0)	
*Clinical outcome*				
Hospital stay, days		5.2 ± 2.5	5.9 ± 3.2	0.293
Mortality		1/25 (4.0)	13/110 (13.4)	0.466
Recurrence		1/25 (4.0)	10/110 (10.4)	0.689
Follow-up duration, years		2.3 [1.0, 4.6]	2.6 [1.2, 4.2]	0.576

Data are expressed as *n* (%) or mean ± standard deviation

Data in the last row are expressed as “Mortality, *p* = 0.466; Recurrence, *p* = 0.689”

*BMI* body mass index, *COPD* chronic obstructive pulmonary disease, *DLCO* diffusing capacity of the lung for carbon monoxide, *EGFR* epidermal growth factor receptor, *FEV*_*1*_ forced expiratory volume in 1 s, *FVC* forced vital capacity, *LUDS* left upper division segmentectomy, *LUL* left upper lobectomy, *NSCLC* non-small cell lung cancer, *LVI* lymphovascular invasion, *VA* alveolar volume, *VATS* video-assisted thoracoscopic surgery, *VPI* visceral pleural invasion

Both groups showed similar preoperative PFT results and pathological stages after PSM (Table 2). During the postoperative period, patients who had persistent cough and required cough medication for at least 3 weeks were more commonly observed in the LUL group (*p* = 0.001).

**Table 2:** Clinical characteristics of patients with left-side NSCLC according to the extent of surgical resection after propensity score matching

Variables	LUDS [*n* = 25]	LUL [*n* = 25]	*p* value
Age, years	62.1 ± 12.0	64.5 ± 12.0	0.500
Female	16 (64)	10 (40)	0.101
BMI, kg/m^2^	25.1 ± 4.5	24.8 ± 3.8	0.307
FEV_1_, L	2.36 ± 0.69	2.50 ± 0.56	0.462
FEV_1_/FVC, %	71.9 ± 10.9	72.3 ± 7.65	0.869
DLCO, %	99.3 ± 17.7	98.9 ± 17.5	0.932
Tumor size, cm	1.6 ± 1.3	2.6 ± 1.6	0.063
Pathologic stage			0.508
Stage I	20 (80)	18 (72)	
Stage II	5 (20)	7 (28)	
CTD indwelling time, days	4.5 ± 3.8	4.8 ± 3.4	0.598
*Outpatients records review*			
Persistent coughing^a^	4 (16)	15 (60)	0.001
Dyspnea^b^	2 (8)	4 (16)	0.667
Pneumonia^c^	2 (4)	2 (8)	1.000
Readmission within 30 days	2 (4)	3 (4)	0.667

Data are expressed as *n* (%) or mean ± standard deviation

*BMI* body mass index, *CTD* chest tube drainage, *DLCO* diffusing capacity of the lung for carbon monoxide, *FEV*_*1*_ forced expiratory volume in 1 s, *FVC* forced vital capacity, *LUDS* left upper division segmentectomy, *LUL* left upper lobectomy, *NSCLC* non-small cell lung cancer

^a^Patients who have prescribed medication for coughing over postoperative 3 weeks after excluding other pulmonary causes

^b^Patients who consulted for exertional or resting dyspnea and were excluded for cardiogenic causes

^c^Patients who were prescribed for antibiotics regarding pneumonia management over 2 weeks

### Lung Volume and Bronchial Tortuosity Measurement

Pre- and postoperative lung volumes are shown in Table 3. The preoperative left-sided lung volume did not differ between the two groups (LUL, 1949.9 mL; LUDS, 1978.4 mL; *p* = 0.795); however, postoperative left-sided lung volume was significantly preserved in the LUDS group (*p* = 0.006). After surgery, the LUDS group had a significantly smaller decrease in left-side lung volume compared with that in the LUL group (LUDS, 323.6 ± 521.4 mL; LUL, 690.7 ± 332.8 mL; *p* = 0.004). The right-sided volume did not differ significantly between the groups.

**Table 3:** Pulmonary function/volume and bronchial tortuosity results according to the extent of surgical resection after propensity score matching

Variables	LUDS [*n* = 25]	LUL [*n* = 25]	*p* value
Pulmonary function test			
Preoperative FVC, L	3.33 ± 0.871	3.41 ± 0.853	0.748
Postoperative FVC, L	2.81 ± 0.694	2.79 ± 0.488	0.938
Preoperative FEV_1_, L	2.42 ± 0.659	2.43 ± 0.638	0.960
Postoperative FEV_1_, L	2.05 ± 0.526	1.87 ± 0.302	0.412
Preoperative FEV_1_, %	98.76 ± 19.34	95.8 ± 18.44	0.598
Postoperative FEV_1_, %	73.38 ± 8.766	67.88 ± 9.702	0.254
Preoperative DLCO, %	97.7 ± 20.40	101.4 ± 14.96	0.490
Postoperative DLCO, %	81.71 ± 13.97	73.6 ± 9.659	0.291
Lung volume measurements			
Right			
Preoperative, mL	2284.5 ± 453.6	2392.6 ± 478.7	0.235
Postoperative, mL	2470.7 ± 544.5	2460.9 ± 500.5	0.932
Volume difference, mL	108.1 ± 444.0	112.4 ± 499.3	0.788
Left			
Preoperative, mL	1978.4 ± 539.1	1949.9 ± 392.1	0.795
Postoperative, mL	1585.1 ± 493.0	1261.5 ± 273.0	0.006
Volume difference, mL	323.6 ± 521.4	690.7 ± 332.8	0.004
LMB CI			
Preoperative CI	1.032 ± 0.170	1.033 ± 0.080	0.810
Postoperative CI	1.074 ± 0.035	1.097 ± 0.036	0.013
CI difference	0.042 ± 0.036	0.065 ± 0.032	0.027

Data are expressed as mean ± standard deviation

*CI* curvature index, *DLCO* diffusing capacity of the lung for carbon monoxide, *FEV*_*1*_ forced expiratory volume in 1 s, *FVC* forced vital capacity, *LMB* left main bronchus, *LUDS* left upper division segmentectomy, *LUL* left upper lobectomy

The left main bronchus-curvature index (LMB-CI) was comparable between the two groups preoperatively, but postoperative LMB-CI was higher in the LUL group (LUDS, 1.074 ± 0.035; LUL, 1.097 ± 0.036; *p* = 0.013), and the difference between the preoperative and postoperative period was also substantial in the LUL group (LUDS, 0.042 ± 0.036; LUL, 0.065 ± 0.032; *p* = 0.027).

## Discussion

Thoracic surgeons have investigated bronchial deviations following pulmonary resection. Recent advancements in radiological techniques have facilitated the visual measurement of the bronchial angle via a three-dimensional analysis. Several studies have identified changes in the bronchial angle following lobectomy and correlated them with clinical outcomes and pulmonary function results. However, the clinical impact of the lingular segment in left upper lobe lung cancer has not been described previously; therefore, we compared the tortuosity of the bronchus between the propensity score-matched LUDS and LUL groups and assessed its correlation with clinical respiratory symptoms. Our findings suggested that patients who underwent LUL had significantly higher bronchial angulation and a higher incidence of persistent cough, even though both the LUL and LUDS groups had similar lung volumes as measured by PFT and chest CT. Collectively, these findings provide novel insights into the clinical implications of preserving the lingular segment of the lung.

The use of segmentectomy across the two segments in preserving pulmonary function has been strongly debated. Nomori et al.^12^ evaluated perioperative pulmonary function using single-photon emission CT and reported that LUDS can result in a marked systemic and lobar decrease in lung function, similar to LUL. Our study yielded similar results in terms of FEV_1_. This may have been attributed to the relatively larger size of the LUD segments and the postoperative decrease in the lingular segment, which was comparable with right middle lobe syndrome after right upper lobectomy. However, FEV_1_ or spirometry parameters may not comprehensively represent postoperative pulmonary function. Spirometry results are based on a comparison with the healthy population^13^ and therefore do not reflect the impact of postoperative changes in the thoracic cavity and structure. Hence, measuring the bronchus angle may be an additional method for evaluating postoperative bronchopulmonary function.

In this study, the tortuosity index was determined by measuring the bronchial angulation via LMB-CI, which is a newly developed approach for measuring bronchial angulation.^11^ Previous studies have used CT reconstruction^9^ or coronal images of the bronchus bifurcation^10^ to evaluate the bronchus angle. In comparison, our approach to measuring bronchial tortuosity should be more objective because the midline curved and straight lengths are automatically reconstructed, thereby facilitating the reproducibility of the results. Similar to the findings of Lu et al., with respect to the correlation between the bronchial angle and postoperative intractable cough, we also observed a significant increase in persistent cough among patients with a higher LMB-CI. Approaches that measure bronchial angulation, such as LMB-CI, should be further investigated and standardized in cases where PFT parameters do not demonstrate mild lung function changes or clinical implications.

This study had several limitations. First, the study had a retrospective design and a small sample size, which may have led to bias in the results. As we did not assess patients’ respiratory symptoms with questionnaires in advance, there might be patients who have symptoms but did not report this. Although we reviewed medical records and prescriptions for cough depressant, it might not be sufficient to overcome this type of bias. Second, differences in tumor characteristics, such as size and proportion of ground-glass opacities, between the two groups could have been confounding factors in the analysis of postoperative respiratory symptoms. Other factors, such as the surgical approach (video-assisted thoracic surgery or thoracotomy), pain management protocols, and different cardiopulmonary functions, could have influenced long-term clinical outcomes. Advances in surgical techniques and standardized protocols for the postoperative care of lung cancer patients over time may have also interfered with the clinical results. Finally, the impact of different lymph node dissection techniques was not integrated into the analyses. Future studies with larger sample sizes and more controlled designs are warranted to obtain more conclusive results.

## Conclusion

LUDS may be a promising option for preventing marked bronchial angulation and decreasing postoperative persistent cough in patients with left upper lobe lung cancer. Although PFT parameters did not demonstrate the benefits of LUDS over LUL, LUDS should be considered for preserving the normal bronchus anatomy and parenchymal volume. Further research is required to establish a standardized approach for measuring bronchial structures and postoperative lung volume to provide a more comprehensive evaluation of postoperative lung function.

## Data Availability

The data underlying this study will be shared by the corresponding author upon request.
